# TetraMabs: simultaneous targeting of four oncogenic receptor tyrosine kinases for tumor growth inhibition in heterogeneous tumor cell populations

**DOI:** 10.1093/protein/gzw037

**Published:** 2016-09-26

**Authors:** Raffaella Castoldi, Jürgen Schanzer, Christian Panke, Ute Jucknischke, Natalie J. Neubert, Rebecca Croasdale, Werner Scheuer, Johannes Auer, Christian Klein, Gerhard Niederfellner, Sebastian Kobold, Claudio Sustmann

**Affiliations:** 1 pRED, Roche Pharma Research & Early Development, Roche Innovation Center, Munich, Germany; 2 pRED, Roche Pharma Research & Early Development, Roche Large Molecule Research, Roche Innovation Center, Munich, Nonnenwald 2, 82377 Penzberg, Germany; 3 pRED, Roche Pharma Research & Early Development, Roche Innovation Center, Zuerich, Switzerland, Wagistrasse 18, 8952 Schlieren; 4 Center of Integrated Protein Science Munich (CIPS-M) and Division of Clinical Pharmacology, Department of Medicine IV, Klinikum der Universität München, Lindwurmstraße 2a, 80337 Munich, Germany, Member of the German Center for Lung Research (DZL)

**Keywords:** cancer, cMet, EGFR, HER3, IGF1R, receptor tyrosine kinase, tetraspecific antibody, therapeutic antibody

## Abstract

Monoclonal antibody-based targeted tumor therapy has greatly improved treatment options for patients. Antibodies against oncogenic receptor tyrosine kinases (RTKs), especially the ErbB receptor family, are prominent examples. However, long-term efficacy of such antibodies is limited by resistance mechanisms. Tumor evasion by *a priori* or acquired activation of other kinases is often causative for this phenomenon. These findings led to an increasing number of combination approaches either within a protein family, e.g. the ErbB family or by targeting RTKs of different phylogenetic origin like HER1 and cMet or HER1 and IGF1R. Progress in antibody engineering technology enabled generation of clinical grade bispecific antibodies (BsAbs) to design drugs inherently addressing such resistance mechanisms. Limited data are available on multi-specific antibodies targeting three or more RTKs. In the present study, we have evaluated the cloning, eukaryotic expression and purification of tetraspecific, tetravalent Fc-containing antibodies targeting HER3, cMet, HER1 and IGF1R. The antibodies are based on the combination of single-chain Fab and Fv fragments in an IgG1 antibody format enhanced by the knob-into-hole technology. They are non-agonistic and inhibit tumor cell growth comparable to the combination of four parental antibodies. Importantly, TetraMabs show improved apoptosis induction and tumor growth inhibition over individual monospecific or BsAbs in cellular assays. In addition, a mimicry assay to reflect heterogeneous expression of antigens in a tumor mass was established. With this novel *in vitro* assay, we can demonstrate the superiority of a tetraspecific antibody to bispecific tumor antigen-binding antibodies in early pre-clinical development.

## Introduction

Receptor tyrosine kinases (RTKs) are prominent targets in tumor cell biology. Fifty-eight human RTKs falling in 20 subfamilies are known and for many of them it has been shown that aberrant activation promotes growth, apoptosis resistance and metastasis of tumor cells (Lemmon and Schlessinger, 2010). Among the ErbB family of proteins, ErbB1/EGFR/HER1, ErbB2/HER2 and ErbB3/HER3 are most actively pursued as targets in the clinic. Focusing only on antibody-based therapies, four therapeutic antibodies, cetuximab and panitumumab for EGFR/HER1 and trastuzumab alone or in combination with pertuzumab for HER2, are approved for treatment of different solid tumors (Ocana and Pandiella, 2013). At least six different HER3 antibodies are in pre-clinical and clinical development but none has been approved yet (Aurisicchio *et al*., 2012; Temraz *et al*., 2016).

Despite initial efficacy, resistance formation is usually limiting long-term application of ErbB-targeted drugs (Hynes and Lane, 2005; Patel and Leung, 2012). ErbB receptor activation depends on homo- or heterodimerization of receptors in a ligand-dependent—15 different ligands have been described so far—or ligand-independent manner (Oda *et al*., 2005). HER3 signaling is mediated via heterodimers as the tyrosine kinase domain is inactive (Jura *et al*., 2009). ErbB crosstalk, e.g. switching from HER1/HER3 to HER2/HER3 mitogenic signaling, is one potent mediator of such resistance (Yonesaka *et al*., 2011; Noto *et al*., 2013). It has been shown that inhibition of two ErbB family members can be more efficacious than single inhibition (Tebbutt *et al*., 2013). Examples are MEHD7945A and MM-111. MEHD7945A, a HER1/HER3-targeting bispecific antibody, is currently in clinical evaluation (Schaefer *et al*., 2011; Huang *et al*., 2013). Development of MM-111, a bispecific antibody targeting HER2/HER3, has been terminated upon lack of clinical efficacy (McDonagh *et al*., 2012). Beyond ErbB, there is now sound evidence that other RTK can compensate for the loss of ErbB-mediated signaling as the downstream signaling pathways are related (Patel and Leung, 2012). Furthermore, RTKs can be grouped in different classes, which preferentially compensate each other upon inhibition of a member of this group (Wagner *et al*., 2013).

It has been shown that cMet can mediate resistance to HER1 inhibition (Karamouzis *et al*., 2009). More recently, the scope of this interplay has been addressed in a number of cancer cell lines and various other proteins have been found to be part of this signaling network (Gusenbauer *et al*., 2013). Still, HER1 and cMet seem to be the central and essential players of this network (Guo *et al*., 2008). We have recently published a study on a bispecific HER1- and cMet-targeting antibody, which inhibits receptor crosstalk (Castoldi *et al*., 2013). Similarly, IGF1R can counteract inhibition of HER1 and render monospecific inhibition futile (Hendrickson and Haluska, 2009). These findings have already led to the generation of bispecific antibodies (BsAbs) targeting HER1 and IGF1R in various formats such as a tetravalent single-chain Fv (scFv)-based IgG1 antibody (Croasdale *et al*., 2012) and a bivalent one arm single-chain Fab (scFab) IgG1 antibody enhanced by the knob-into-hole technology (Schanzer *et al*., 2014).

The basic principle of inhibiting two or more prominent signaling pathways simultaneously relies on the assumption that at some point tumor cells cannot cope fast enough with the loss of pro-survival signaling relayed by RTKs and will subsequently undergo apoptosis. Next generation multi-specific antibodies may target several RTK simultaneously to pre-emptively address resistance events. In addition, multi-specific antibodies have the capability to simultaneously target *a priori* heterogeneous cell populations. We already explored in the past the properties of trispecific, trivalent Fc-containing antibodies-targeting RTKs and could demonstrate that such constructs can be produced, are non-agonistic and inhibit tumor growth in a similar fashion as the combination of parental antibodies (Castoldi *et al*., 2012).

In the current study, we extend this approach to tetraspecific, tetravalent scFab and scFv-based Fc-containing knob-into-hole antibodies, which can simultaneously bind to four different RTKs, namely HER1, HER3, IGF1R and cMet for the aforementioned reasons of receptor crosstalk and resistance. Apart from the characterization of these antibodies, one major aim of this study is to demonstrate the advantage of tetraspecific antibodies in comparison to BsAbs, which are now emerging in clinical trials. In particular, besides demonstrating efficacy on tumor cells expressing more than one of the targeted RTKs, we established a mimicry assay of tumor heterogeneity in which the heterogeneous target expression in between cells in a tumor mass was modeled by mixtures of lung cancer tumor cells. Here, we demonstrate that the presented tetraspecific antibodies can be produced in eukaryotic cells, are fully functional, non-agonistic and in all *in vitro* cellular models superior to BsAbs targeting either HER3 and cMet or HER1 and IGF1R.

## Materials and methods

### Cell culture

MDA-MB-175-VII, BxPC3, H441 and A549 were obtained from ATCC; H322M from the NCI and H596 from Chugai Pharmaceuticals Co., Ltd. All cell lines were cultured in RPMI 1640 medium with 10% fetal calf serum (FCS), non-essential amino acids, sodium pyruvate and l-glutamine (Gibco). Cells were propagated according to standard cell culture protocols.

### Antibodies and reagents

TsAb2v2 and TsAb3v1 were generated by cloning variable heavy- and light-chain domain sequences extracted of the corresponding HER1, HER3, IGF1R and cMet parental antibodies in mammalian expression vectors as described previously (Castoldi *et al*., 2012; Schanzer *et al*., 2014). For TsAb2v2, HER1 and IGF1R sequences were cloned as scFabs in the order V_L_-C_L_-(G_4_S)_6_GG-V_H_-C_H1_. In the case of TsAb3v1, only the IGF1R Fab arm was designed as scFab and the HER1-binding arm is a disulfide stabilized light and heavy chain. HER3 and cMet scFvs were cloned as V_H_-(G_4_S)_3_-V_L_ fusion at the C-terminus of the respective CH3 domain via a (G_4_S)_2_ connector. The expression vectors were co-transfected in HEK293 cells (Life Technologies) and purified as previously described (Metz *et al*., 2011). Homogeneity of the final products was evaluated with an Agilent HPLC 1100 (Agilent Technologies) using a TSK-GEL G3000SW size exclusion chromatography (SEC) column (Tosoh Corp.). HER1, IGF1R, cMet and HER3 receptor extracellular domains were cloned in eukaryotic expression vectors, transiently expressed in HEK293 and purified by affinity purification. Recombinant huHGF, huIGF (R&D Systems) and huEGF (Gibco), and huHeregulin-β1 (Peprotech) were purchased. The antibodies pHER1, pAKT1 (Epitomics), HER1 (Millipore), pHER3, pIGF1R, pcMet, cMet, pMAPK, MAPK, AKT (CST), HER3, IGF1R (Santa Cruz) and β-actin (Abcam) were purchased. Human IgG was received from Jackson ImmunoResearch (JIR).

### Surface plasmon resonance

All surface plasmon resonance (SPR) experiments were performed using a Biacore T200 instrument with running buffer PBS 0.05% (w/v) Tween20 and dilution buffer prepared with additional supplement of 1 mg/mL BSA. Standard amine coupling was performed according to the manufacturer's instructions. For the kinetic profile determination, antibodies under analysis were captured via an α-human Fc-Ab (Jackson ImmunoResearch). CM5 Chip was used for monomeric HER1, cMet and HER3, C1-Chip was used to reach monovalent binding of the dimeric IGF1R. α-human Fc-Ab was amine coupled with a density of ~1000 RU for the CM5-Chip and 200 RU for the C1-Chip. Capture levels of antibodies were ~40–70 and 5–10 RU, respectively. Five increasing concentrations of each of the receptors were injected at a flow rate of 50 µl/min for 180 seconds association time and dissociation of 1800 up to 600 seconds, depending on the *k_d_*-rate constants of each receptor at 37°C. To demonstrate, reproducibility duplicates of at least one receptor concentration were analyzed with following receptor concentration ranges: cMet *c* = 500–6.17 nM, IGF1R *c* = 133.3–1.65 nM, HER3 *c* = 333.3–4.12 nM, HER1 *c* = 300–3.7 nM. Final regeneration was performed after each cycle using 10 mM Glycine pH 1.75. Kinetic constants were evaluated by fitting the association and dissociation phase of the analyzed interaction with a Langmuir 1:1 binding model (RI = 0) using usual double referencing (FC1 reference surface with immobilized capture molecule and receptor *c* = 0 nM) by Scrubber SW V2.0c. For the evaluation of simultaneous binding, the tetraspecific antibodies were injected over amine-coupled HER1 (CM5-Chip, 2000 RU ligand density) and the three remaining receptors were injected consecutively to the already built HER1-antibody-complex using a dual inject followed by a further inject, with contact time 180 seconds each and flow rate 30 µl/min. A temperature of 25°C was chosen to minimize dissociation. Receptors were injected at the following concentrations: cMet *c* = 800 nM, IGF1R *c* = 100 nM, HER3 *c* = 300 nM in different orders. After each cycle, regeneration was performed using 15 mM NaOH.

### Immunoblot

H322M (0.8 × 10E6 cells), BxPC3 (0.7 × 10E6 cells) and MDA-MB-175-VII (1 × 10E6 cells) were seeded in each well of a six-well plate in 0.5% FCS medium and subjected the following day to a 30 min treatment with 0.07 μM of TsAb2v2, TsAb3v1, cMet/HER3 and HER1/IGF1R BsAb, all single parental 5D5 anti-cMet and HER1, IGF1R, HER3 MAbs simultaneously, prior to stimulation with the growth factors (GFs)—50 ng/ml IGF, 50 ng/ml EGF, 500 ng/ml Heregulin, 30 ng/ml HGF. After 10 min of incubation at 37°C, cells were washed with PBS supplemented with 1 mM Na3VO4 and lysed. Immunoblots were performed according to standard protocols. At least three biological replicates were performed.

### Apoptosis assay

H322M (2500 cells/well) were seeded in a 96-well plate 24 hours before treatment. Samples were treated with 0.2 μM TsAb2v2, TsAb3v1, HER1/IGFR BsAb, cMet/HER3 BsAb and parental antibodies (5D5 anti-cMet, HER1, IGF1R and HER3 MAbs) in combination or individually and pre-incubated for 15 min before addition of HGF (30 ng/ml). Apoptosis was evaluated using a Caspase 3/7 Glo luminescent assay (Promega) after 2 days. Staurosporine 10 μM was added 3 hours before reading and used as positive control. Cells treated with an unspecific huIgG antibody were used as negative control. All experiments were done in technical triplicates and at least biological replicates.

### Proliferation assays

MDA-MB-175-VII (10 000 cells/well) and H322M (2500 cells/well) were seeded in a 96-well plate in 10% FCS medium for 24 hours and treated with 0.2 μM of the described antibodies for 15 min before stimulation with HGF 30 ng/ml. Viability was measured in a Cell Titer Glo assay 5 days after treatment, according to the manufacturer's procedures (Promega). For the co-culture experiments A549 (1000 cells/well), H441 (2000 cells/well), H596 and H322M (2500 cells/well) were seeded individually or in proportional combinations of two, three and four cell types and treated as described above. All experiments were done in technical triplicates and biological replicates.

### Affymetrix analysis

Total RNA was isolated from cells using a RNeasy Mini Kit (Qiagen) and cDNA synthesis was carried out using a cDNA synthesis kit (Roche Applied Science). Double-stranded cDNA was purified with a Microarray Target Purification Kit (Roche Applied Science) and transcribed into cRNA using the Roche Microarray RNA Target Synthesis Kit (T7) (Roche Applied Science). The cRNA was subsequently purified with RNeasy Mini-Spin Columns (RNeasy Mini Kit, Qiagen). All procedures were performed according to the manufacturers’ instructions. Around 20 µg of the purified cRNA were fragmented in a total volume of 40 µl making use of a fragmentation buffer (200 mM Tris-acetate, pH 8.1; 500 mM potassium acetate, 150 mM magnesium acetate) at 94°C for 35 min. Around 30 µl of fragmented cRNA solution were mixed with control oligonucleotide, staggered control cRNAs, herring sperm DNA, acetylated BSA and hybridization buffer in a total volume of 300 µl and the hybridization mix was loaded onto Affymetrix Human Genome U133 Plus 2.0 arrays and incubated in a roller device for 16 hours. Finally, the hybridization solution was removed and the arrays were washed and stained as recommended by Affymetrix before being scanned with an Affymetrix GeneChip Scanner 3000 (7G). All samples were measured in triplicates. Data were analyzed using in-house software.

## Results

### Generation of tetraspecific antibody constructs binding HER1, IGF1R, HER3 and cMet

**Fig. 1:**
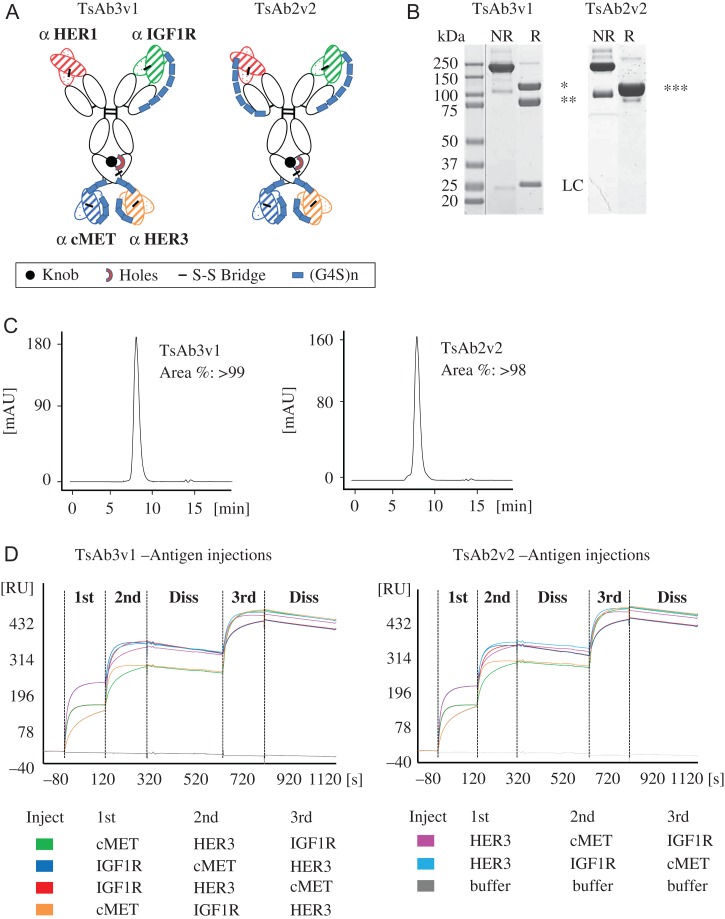
TsAb3v1 and TsAb2v2 antibody generation. (**A**) Schematic representation of the two TsAb constructs. cMet and HER3 V_H_-(G_4_S)_3_-V_L_ scFvs were fused to the C-terminus of ‘knob-into-hole’ heavy chains of an IgG1 antibody backbone by a (G_4_S)_2_ connector. N-terminal scFabs targeting HER1 and IGF1R were designed as V_L_-C_L_-(G_4_S)_6_GG-V_H_-C_H1_ in the TsAB2v2 construct. TsAb3v1 consists of an IGF1R targeting one arm scFab and a disulfide stabilized Fab binding to HER1. (**B**) SDS-PAGE analysis of purified TsAb3v1 and TsAb2v2. (**C**) Analytical SEC of purified TsAb3v1 and TsAb2v2. (**D**) SPR analysis of simultaneous binding of TsAb3v1 and TsAb2v2 to HER1, HER3, IGF1R and cMet. A color code of the injection order of the extracellular receptor domains of IGF1R, HER3 and cMet on the tetraspecific antibodies bound to a HER1-coated CM5 chip is depicted below the graphs.

Two novel tetraspecific antibody formats TsAb2v2 and TsAb3v1 with antigen-binding specificities for HER1, IGF1R, HER3 and cMet were generated. A scFab format was employed to prevent light-chain mismatch of the IGF1R and HER1-binding Fab arms at the N-terminus of the human IgG1 backbone as described previously (Schanzer *et al*., 2014). Using either one or two scFab arms was the only difference between the two tetraspecific antibody formats. The antibody Fc region was further engineered by the knobs-into-holes technology to enable addition of two different scFv (Ridgway *et al*., 1996). HER3 and cMet-binding scFvs were joined by a G4S linker and fused at the C-terminus of the CH3 domain to generate tetraspecific antibodies (Fig. 1A).

Both molecules were transiently expressed in HEK293 suspension cells with a preliminary yield of 12.4 and 19.1 μg/ml for TsAb2v2 and TsAb3v1, respectively. Purification was performed by standard procedures using Protein A affinity chromatography followed by SEC. The final products (yield 7.4 μg/ml for TsAb2v2 and 16.0 μg/ml for TsAb3v1) were characterized by SDS-PAGE and analytical HPLC showing a high degree of purity with a product homogeneity >98% (Fig. 1B and C). Both constructs were able to bind HER1, HER3, IGF1R and cMet with nM affinities comparable to the parental monospecific antibodies as assessed by SPR (Table I, Supplementary Fig. 1). IGF1R is a disulfide bridged homo-dimer. Off-rate fits were imprecise and are associated with its homo-dimeric nature and bivalent or rebinding events (Supplementary Fig. 1). This was previously evaluated in more detail for trispecific antibodies (Castoldi *et al*., 2012). To address simultaneous binding of the tetraspecific antibodies to all four target antigens, the recombinant extracellular domains of HER3, IGF1R and cMet were sequentially injected in different orders on tetraspecific antibodies bound to a HER1-coated chip surface and binding was demonstrated at each injection point by SPR analysis (Fig. 1D). In summary, we were able to express the molecules in HEK293 cells and generate a homogeneous preparation of the tetraspecific antibodies. TsAb2v2 and TsAb3v1 were binding to the extracellular receptor domains with similar affinities as the parental antibodies and able to simultaneously form a complex with all four target antigens.

**Table I.** Kinetic constants for binding of extracellular domains of cMet, HER3, HER1 and IGF1R receptors to TsAb3v1, TsAb2v2 and parental antibodies 5D5 anti-cMet, HER3 MAb, HER1 MAb, IGF1R MAb

Antibody	Analyte	[M^−1^·s^−1^]	[s^−1^]	[min]	[M]
TsAb3v1	cMet	4.1E+04 ± 0.2%	5.0E−04 ± 0.4%	23.3	1.2E−08 ± 0.6%
TsAb2v2	cMet	4.1E+04 ± 0.2%	5.4E−04 ± 0.4%	21.6	1.3E−08 ± 0.5%
5D5	cMet	6.3E+04 ± 0.2%	2.7E−04 ± 0.4%	43.4	4.2E−09± 0.5%
TsAb3v1	HER3	8.3E+04 ± 0.2%	7.9E−04 ± 0.1%	14.6	1.0E−08 ± 0.3%
TsAb2v2	HER3	6.9E+04 ± 0.1%	9.3E−04 ± 0.1%	12.5	1.3E−08 ± 0.2%
Mab HER3	HER3	1.7E+05 ± 0.1%	6.8E−04 ± 0.1%	17.1	4.0E−09 ± 1.7%
TsAb3v1	EGFR	9.0E+04 ± 0.2%	4.6E−04 ± 0.4%	25.1	5.1E−09 ± 0.4%
TsAb2v2	EGFR	9.0E+04 ± 0.2%	5.1E−04 ± 0.4%	22.6	5.7E−09 ± 0.4%
Mab HER1	EGFR	8.3E+04 ± 0.2%	6.1E−04 ± 0.3%	18.8	7.4E−09 ± 0.4%
TsAb3v1	IGF1R	7.9E+05 ± 3.8%	4.8E−03 ± 1.9%	2.4	6.1E−09 ± 4.9%
TsAb2v2	IGF1R	9.4E+05 ± 3.2%	4.5E−03 ± 1.5%	2.6	4.8E−09 ± 4.2%
Mab IGF1R	IGF1R	1.1E+05 ± 1.9%	2.7E−03 ± 0.7%	4.3	2.5E−09 ± 1.6%

### Inhibition of RTK activation and apoptosis induction in tumor cells

**Fig. 2:**
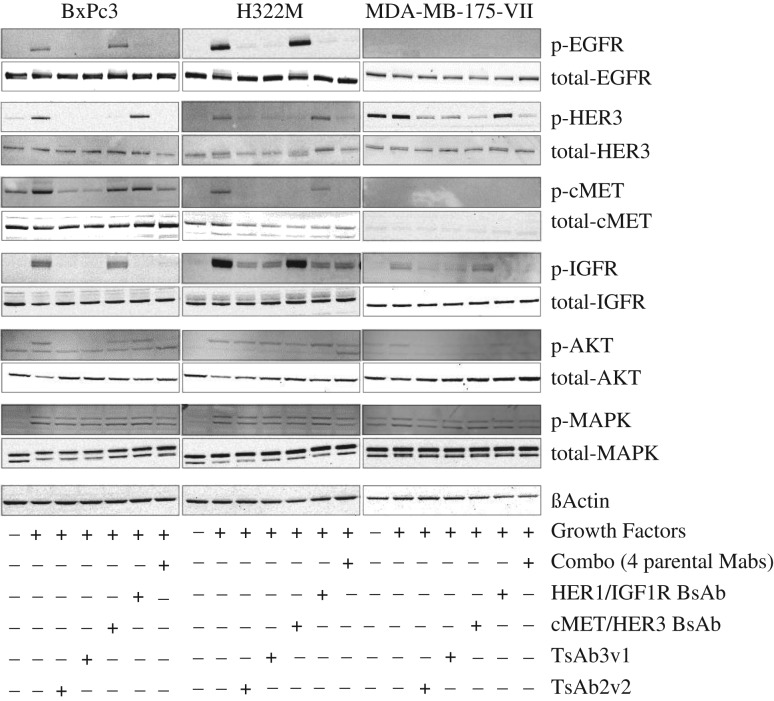
Immunoblot analysis of TsAb3v1 and TsAb2v2 effect on signaling in BxPC3, H322M and MDA-MB-175-VII cells. Expression and phosphorylation status of HER1, HER3, cMet, IGF1R, AKT and MAPK in cells after a 30 minutes incubation with 0.07 µM TsAb3v1, TsAb2v2, BsAb HER1/IGF1R, BsAb cMet/HER3, and the combination of all four parental antibodies. Following the antibody incubation, cells were stimulated with the relative growth factors EGF, Heregulin, HGF and IGF1 for 10 minutes, lysed and subjected to immunoblotting. Tetra-, bi- and monospecific antibody molecules or combinations corresponding to expression and phosphorylation status of RTK are shown as ± symbols below the graph.

We initially addressed the presence and activation status of the four RTK in the tumor cell lines MDA-MB-175-VII, BxPC3 and H322M by immunoblotting. For *in vitro* receptor activation, the medium was supplemented with a mixture of the growth factors EGF, HRG, HGF and IGF to compensate for the absence of tumor–stroma interactions. Basal and ligand-dependent activation was determined by receptor phosphorylation status. Downstream signaling occurs predominantly via the MAPK and PI3K signaling pathways. BxPC3 and H322M displayed robust activation of all four RTKs as determined by receptor phosphorylation while MDA-MB-175-VII was only responding to IGF and HRG treatment by activation of the respective receptors (Fig. 2). MAPK as well as PI3K, as measured via phosphorylated AKT, were activated in all instances in which GF were added. We next asked the question if treatment with individual parental antibodies, a combination thereof or the tetraspecific antibodies (i) impairs this activation and if (ii) the tetraspecific antibody performs similar to the combination (combo) of all four parental antibodies. Indeed, the tetraspecific antibodies inhibited receptor activation as well as downstream signaling and showed similar activity as the combination of the parental antibodies (Fig. 2). Furthermore, we could not observe any agonistic activity.

**Fig. 3:**
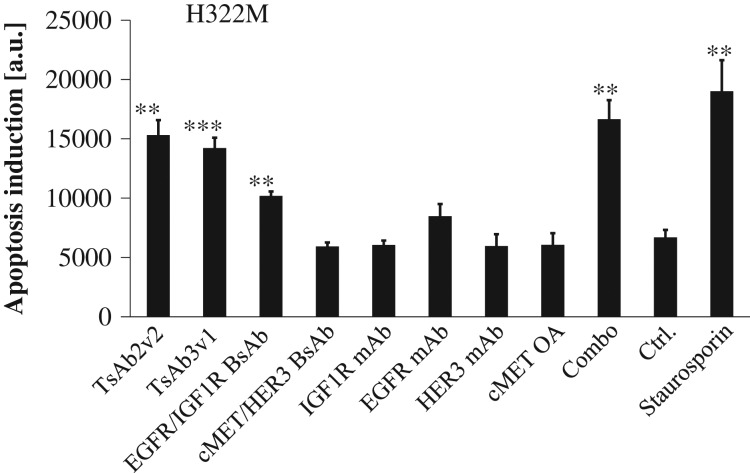
TsAb3v1 and TsAb2v2 mediated tumor cell death. H322M cells were incubated for 2 days with TsAb3v1, TsAb2v2, the BsAbs HER1/IGF1R and cMetHER3 and the combination as well as all four parental monospecific antibodies individually. Apoptosis induction was measured after cell lysis in a Caspase Glo assay and reported as luminescence units. Staurosporine was used as positive control. Statistical significance was calculated versus a negative control (cells treated with an unspecific huIgG antibody); **P < 0.01; ***P < 0.001.

We next asked if treatment with the inhibitory antibodies results in increased apoptosis in tumor cells. Apoptosis induction was followed by measuring Caspase 3/7 activity in H322M cells. As expected, only addition of antibody combinations elicited a significant increase of apoptosis after 2 days of incubation. Both tetraspecific antibodies significantly induced apoptosis at a concentration of 200 nM in this assay, to a similar degree as the combination treatment, which was almost equally potent as incubation with a 10 µM solution of the multi-kinase inhibitor staurosporine (Fig. 3).

### Tetraspecific antibody-mediated inhibition of tumor cell growth

**Fig. 4:**
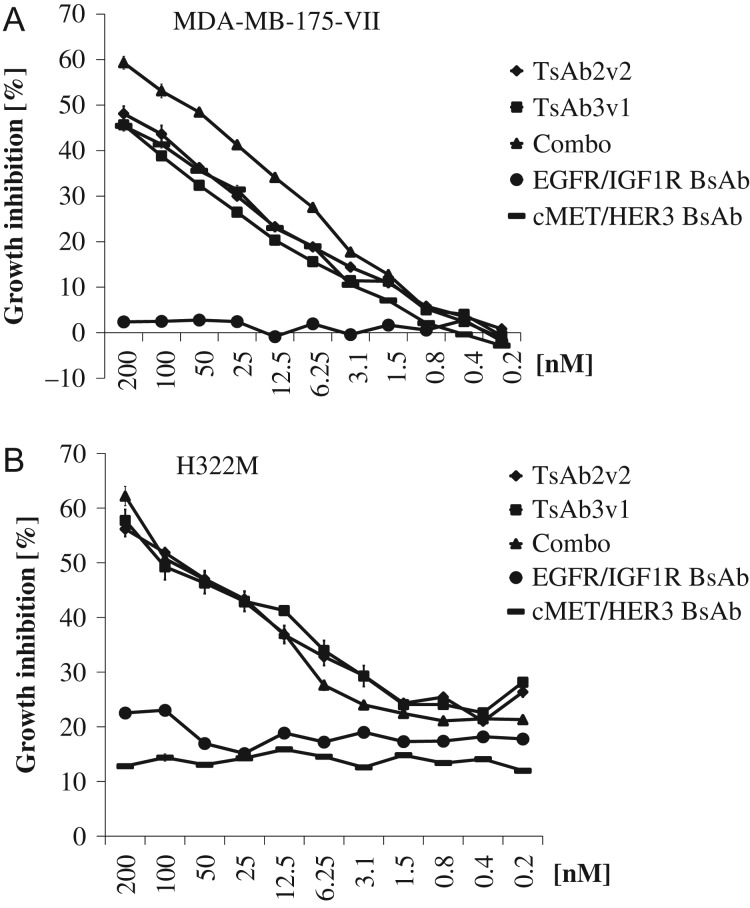
TsAb3v1 and TsAb2v2 tumor cell proliferation inhibition. MDA-MB-175-VII and H322M cells were incubated for 5 days with TsAb3v1, TsAb2v2, the BsAbs HER1/IGF1R and cMetHER3 and the combination of all four parental monospecific antibodies at the reported concentrations. Cell viability was measured in a Cell Titer Glo assay and calculated as growth inhibition versus untreated control.

Inhibition of tumor cell growth was analyzed in MDA-MB-175-VII and H322M cells. Efficacy was evaluated in comparison to the combination of the four parental antibodies and two BsAbs, binding either HER3 and cMet or HER1 and IGF1R. Viability was assessed after 5 days of treatment with a concentration series of the antibodies. In MDA-MB-175-VII cells, TsAb2v2 and TsAb3v1 did not display any additional growth inhibitory effect compared to the BsAb cMet/HER3, while BsAb HER1/IGF1R was completely inactive (Fig. 4A). At the highest concentration growth inhibition reached 50%, and was slightly lower in comparison to the four monospecific antibodies sequestered simultaneously (60% growth inhibition; *P* < 0.01 and *P* < 0.001 significant difference versus TsAb2v2 and TsAb3v1 at 200 nM). Immunoblot data indicate strong activation of the HER3 receptor in MDA-MB-175-VII cells, presumably mediated by HER2 (Fig. 2).

Treatment response in H322M was quite similar to what has been observed in MDA-MB-175-VII with the exception that both BsAbs displayed only a 10–20% basal inhibition, which was not dose dependent (Fig. 4B). Potency of the tetraspecific antibodies was similar to the combination of the parental antibodies (60% growth inhibition at 200 nM) and significantly better than the two BsAbs (*P* < 0.001). These findings confirm the inhibitory activity of the tetraspecific antibodies *in vitro* and indicate that tumor cell growth inhibition by targeting four RTK  simultaneously is more potent than treatment with individual BsAbs.

### Tetraspecific antibodies show increased cellular proliferation inhibition compared to bispecific HER1/IGF1R and cMet/HER3 antibodies in co-cultures of different NSCLC cell lines

All of the RTK targeted by the tetraspecific antibodies have been shown to play a role in non-small cell lung cancer (NSCLC). We thus selected a panel of four NSCLC cell lines to characterize the tetraspecific antibodies in more depth. Tumor heterogeneity is to a large extent dependent on the tumor micro-environment and influenced by presence of extracellular matrix, fibroblasts or distance to blood vessels all of which is challenging to mimic *in vitro* or simplify for mode of action analyses. The aim of this analysis was to establish a more complex *in vitro* environment that could better reflect the tumor heterogeneity observed *in vivo*. The four cell lines A549, H596, H441 and H322M were cultured individually or in combination in groups of two, three and four for 5 days in the presence or absence of the indicated antibodies. Individual growth curves were determined and seeding conditions selected such that each cell line was equally presented in the mixture (data not shown, for cell numbers, see ’Materials and Methods’ section). We hypothesized that escape and clonal outgrowth of tumor cells upon targeted inhibition of two or more receptors will be better observable in complex mixtures of tumor cells.

**Fig. 5:**
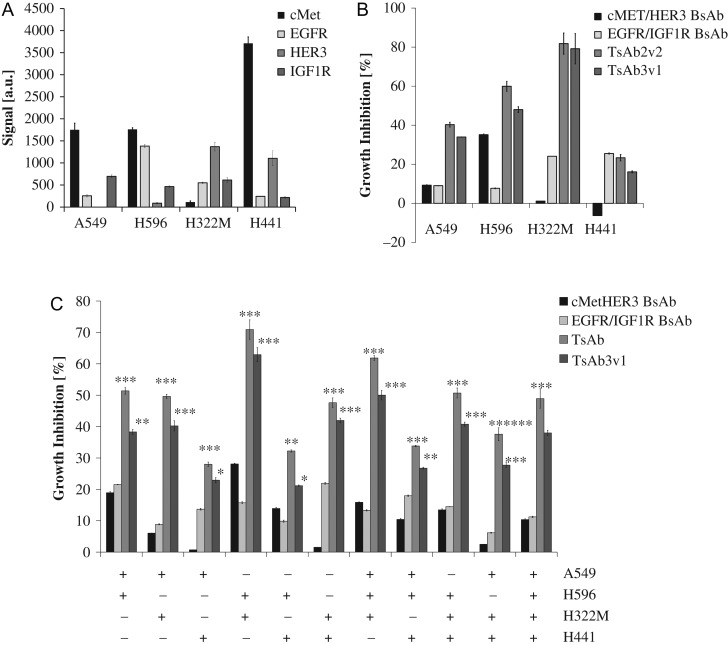
TsAb3v1 and TsAb2v2 proliferation inhibition of NSCLC co-cultured cell lines. (**A**) RNA expression of cMet, HER1, HER3 and IGF1R in A549, H596, H322M and H441 cells measured by affymetrix analysis. (**B**) Percentage growth inhibition versus control of A549, H596, H322M, H441 cells grown individually and treated for 5 days with cMetHER3 BsAb, HER1/IGF1R BsAb, TsAb3v1 and TsAb2v2. Cell viability was evaluated in a Cell Titer Glo assay. (**C**) Percentage growth inhibition versus control of A549, H596, H322M, H441 cells cultivated in various combinations (shown as ± symbols below the graph) and treated for 5 days with cMetHER3 BsAb, HER1/IGF1R BsAb, TsAb3v1 and TsAb2v2. Cell viability was evaluated in a Cell Titer Glo assay. Statistical significance was evaluated versus the BsAb showing the strongest growth inhibitory effect; *P < 0.05; **P < 0.01; ***P < 0.001

Additional selection criteria for the cell lines were the relative expression levels of the targets. Presence of target mRNA was confirmed by affymetrix analysis (Fig. 5A). In addition, receptor presence on the cell surface was confirmed by flow cytometry (data not shown). Growth inhibition in dependence of antibody treatment was initially evaluated on each cell line individually and compared to the BsAbs. TsAb2v2 and TsAb3v1 showed higher efficacy in comparison to the BsAbs in A549, H596 and H322M cells (Fig. 5B). A potential synergistic activity of the tetraspecific antibodies was observed in H322M where treatment with the two simultaneously given BsAbs resulted in a significantly (*P* < 0.01) lower effect compared to what was observed with each of the two tetraspecific antibodies (data not shown). Inhibitory activity in H441 was overall limited and tetraspecific antibodies do not add to the effect observed with a HER1/IGF1R BsAb. The extent of inhibition is similar to what has been observed with the TKI afatinib and implies a dependency on HER1-mediated signaling (Chao *et al*., 2015). For H596, we and others had previously shown that a cMet/HER1 BsAb inhibits cellular growth and receptor-mediated signaling (Castoldi *et al*., 2013; Spiess *et al*., 2013). A cMet/HER3 BsAb is in this setting similarly active (Fig. 5B).

In a final series of experiments, different combinations of the tumor cell lines were seeded and potency of tetraspecific antibodies and BsAbs was evaluated as described. In the tumor cell combination experiments, the tetraspecific antibodies were clearly more potent than the BsAbs (Fig. 5C). Potency of the BsAbs seems to plateau at ~20% growth inhibition, while the tetraspecific antibodies reach growth inhibitory activities of up to 50%. Inhibition is even higher in the combination of H596 and H322M (Fig. 5C). These two cell lines also individually responded more strongly to TsAb2v2 and TsAb3v1 (Fig. 5B). The stronger potency was significant across all possible combinations of NSCLC cell lines. Our data suggest that multi-specific antibodies can be more efficacious than BsAbs in an *in vitro* model of a heterogeneous tumor environment, in which target expression often shows a high degree of variability between tumor cells.

## Discussion

In the present study, we report the development and* in vitro* characterization of novel tetravalent tetraspecific antibodies for simultaneous targeting of HER1, HER3, IGF1R and cMet on tumor cells with the potential to overcome resistance mechanisms mediated by compensatory signaling between different RTK signaling pathways. The tetraspecific antibodies TsAb2v2 and TsAb3v1 are composed of a knob-into-hole IgG1 Fc region with one or two N-terminal scFabs and C-terminal scFvs. Both molecules were cloned, transiently expressed in HEK293 cells and purified by Protein A and SEC to final product yields of 7.4  and 16.0 mg/l, respectively. Partially unfolded scFab can lead to multimer formation (Hust *et al*., 2007). Having two scFab arms (TsAb2v2) increased in our hands the propensity of aggregate formation as observed in SEC and titers (data not shown).

Binding to HER1, HER3, IGF1R and cMet was maintained in the novel format and simultaneous binding of all four extracellular receptor domains was demonstrated by SPR analysis. In addition, we have shown inhibition of receptor activation in pancreatic, mammary and lung tumor cells in presence of the growth factors EGF, HRG, HGF and IGF by immunoblotting in absence of any agonistic activity. Inhibition of RTK activation by TsAb2v2 and TsAb3v1 led to induction of apoptosis in H322M lung tumor cells comparable to the combination of the monospecific parental antibodies and clearly improved to individual monospecific or bispecific cMet/HER3 or HER1/IGF1R antibodies. Furthermore, growth inhibition of H322M NSCLC cells mediated by the tetravalent antibodies was similar to the combined parental antibodies and superior compared to the BsAbs. In a mixture of A549, H596, H322M and H441 lung tumor cells with different expression levels of HER1, HER3, IGF1R and cMet similar to the heterogenous cell populations in tumors, TsAb2v2 and TsAb3v1 induced a significantly improved tumor growth inhibition in comparison with the corresponding BsAbs. These data suggest a potential competitive advantage of such formats versus similar antibodies active only towards two receptors in a heterogeneous and more complex setting, adding additional value to the potential of a tetraspecific antibody to pre-emptively address resistance events.

We have previously reported the one arm scFab bispecific antibody format as a robust platform technology for bispecific antibody generation allowing for stable production of clinical grade BsAbs in CHO cells with yields of 2–3 g/l (Schanzer *et al*., 2014, 2016). In this format, heterodimerization of two antibody heavy chains with different binding specificities is achieved by applying the knob-into-hole technology of introducing a knob mutation (T366W) into the CH3 domain of one heavy chain, and three mutations to form a hole (T366S, L368A and Y407V) into the CH3 domain of the second heavy chain (Ridgway *et al*., 1996). Then, we have fused one or two scFabs binding to HER1 and IGF1R joined by an internal 32 amino acid G4S linker to the N-terminus and two HER3 and cMet-binding scFvs connected by a 10 amino acid G4S linker to the C-terminus of the molecule. The generation of scFab and scFvs from existing monospecific antibodies allowed us to rapidly generate a tetraspecific antibody without the need of time and labor intensive optimization by phage display as in other multi-specific formats such as the ‘two-in-one’ antibody technology (Schaefer *et al*., 2011). Generation and characterization of a tetravalent but bispecific antibody targeting FAP and DR5 using ‘two-in-one’ technology has been recently published (Brunker *et al*., 2016).

All parental antibodies for the design of the tetravalent antibodies were selected from antibodies, which currently are or have been in various stages of clinical development. The HER1-binding arm in TsAb2v2 and TsAb3v1 is derived from the glycoengineered HER1-antibody Imgatuzumab (GA201), which was in a Phase II trial in patients with metastatic colorectal cancer. However, clinical development of Imgatuzumab was halted due to the negative outcome of the comparison to cetuximab in combination with FOLFIRI in this study (Bridgewater *et al*., 2015). The HER3 antibody Lumretuzumab (RG7116) was recently reported in a Phase I clinical trial in patients with metastatic or advanced HER3 positive solid tumors (Meulendijks *et al*., 2016). The one armed anti-cMet antibody Onartuzumab (OA-5D5) was evaluated in several Phase II clinical trials and one Phase III study in patients with solid tumors (Morley *et al*., 2015). Finally, the IGF1R binding arm in the tetraspecific antibodies is derived from R1507, which was studied in a Phase II trial in patients with recurrent or refractory osteosarcoma or soft tissue sarcomas (Pappo *et al*., 2014). Recently, clinical development of R1507 has been discontinued due to its limited activity in patients with recurrent or refractory bone and soft tissue sarcomas (Pappo *et al*., 2014). Furthermore, a Phase III trial of onartuzumab in combination with the small molecule HER1 inhibitor erlotinib in advanced NSCLC patients failed to show improved efficacy versus erlotinib alone (Charakidis and Boyer, 2014). All these rather discouraging data from clinical trials of monoclonal antibodies-targeting RTK in tumor patients indicate that it is not sufficient to inhibit only one or two signaling pathways to achieve profound anti-tumor activity.

Targeted inhibition of GF receptors relies on the principle that cells are dependent on pro-proliferative and pro-survival signaling from few signaling pathways (Sharma and Settleman, 2007; Pagliarini *et al*., 2015). Successful initial treatment with monotherapy is often accompanied by regrowth partly conferred by crosstalk between signaling networks. This has been well described for the ErbB pathway but also for cMet and IGF1R (Guo *et al*., 2008; Liu *et al*., 2014; Yamaguchi *et al*., 2014). One approach to overcome the limitations of monoclonal antibody targeted therapy against RTK may thus be the development of antibody mixtures targeting HER1, HER2 and HER3 (Jacobsen *et al*., 2015). Another interesting approach is the generation of bispecific RTK antibodies, which has led to numerous reports of bispecific molecules in pre-clinical and clinical development, e.g. (i) MM-141, a bispecific antibody against IGF1R and HER3, is currently in clinical development (Fitzgerald *et al*., 2014.), (ii) a bispecific dual action Fab-based IgG antibody recognizing HER1 and HER3 simultaneously has been studied in clinical trials (Li *et al*., 2015) or (iii) bispecific cMet-HER1 antibodies (Castoldi *et al*., 2013; Zheng *et al*., 2016).

Here, we present *in vitro* data showing that tetraspecific antibodies-targeting HER1, HER3, IGF1R and cMet can be superior to BsAbs with respect to apoptosis induction and tumor growth inhibition. Data on multi-specific antibodies targeting three or more cell surface antigens are still rarely found (Todorovska *et al*., 2001; LaFleur *et al*., 2013). This is at least in part owed to increasingly complex effects on binding, internalization and signaling as well as hurdles in protein engineering to produce stable molecules with high expression yields. Recently, a four-in-one antibody with superior cancer inhibitory activity against HER1, HER2, HER3 and VEGF through disruption of HER/Met crosstalk was described (Hu *et al*., 2015). Another advantage of tetravalent antibodies for later clinical development could be reduced binding to normal tissues since expression of all four receptors as often the case in tumor cells is required for maximal avidity.

Finally, we have developed an *in vitro* mimicry assay by co-culturing several lung cancer cell lines with various expression levels of HER1, HER3, IGF1R and cMet to address heterogeneous expression of RTKs within tumors. With this novel assay, we were able to demonstrate the superiority of a tetraspecific antibody to BsAbs-targeting RTKs. The clear impact of the format of complex antibodies on their function highlights the need for such predictive functional assays.

In conclusion, this study highlights the competitive advantage of complex antibody formats simultaneously targeting several RTKs as well as the importance of rationale design and detailed pre-clinical analysis to understand the mode of action of potential candidates and minimize the risk of failure in later stages of clinical development.

## Abbreviations

TsAb: tetraspecific antibody
scFab: single-chain Fab
scFv: single-chain Fv

## Supplementary data

Supplementary data are available at *PEDS*online.

Supplementary Data
