# Supplementary material for: TetraMabs: simultaneous targeting of four oncogenic receptor tyrosine kinases for tumor growth inhibition in heterogeneous tumor cell populations
Source: Protein Eng Des Sel. 2016 Sep 26;29(10):467–75. doi: 10.1093/protein/gzw037 (PMC5036864; doi:10.1093/protein/gzw037)
Supplement: Supplementary Data [file supp_29_10_467__index.html]

TetraMabs: simultaneous targeting of four oncogenic receptor tyrosine kinases for tumor growth inhibition in heterogeneous tumor cell populations — TetraMabs: simultaneous targeting of four oncogenic receptor tyrosine kinases for tumor growth inhibition in heterogeneous tumor cell populations — Supplementary Data 

# TetraMabs: simultaneous targeting of four oncogenic receptor tyrosine kinases for tumor growth inhibition in heterogeneous tumor cell populations

## Supplementary Data

Supplementary Data

- Supplementary Data - pdf file
