# Supplementary material for: TetraMabs: simultaneous targeting of four oncogenic receptor tyrosine kinases for tumor growth inhibition in heterogeneous tumor cell populations
Source: Protein Eng Des Sel. 2016 Sep 26;29(10):467–75. doi: 10.1093/protein/gzw037 (PMC5036864; doi:10.1093/protein/gzw037)
Supplement: Supplementary Data [file supp_gzw037_160520_SupplFig1Sustmann.pdf]

Supplement figure 1

A B

TsAb3v1

TsAb2v2

parental MAb

C D

TsAb3v1

TsAb2v2

parental MAb

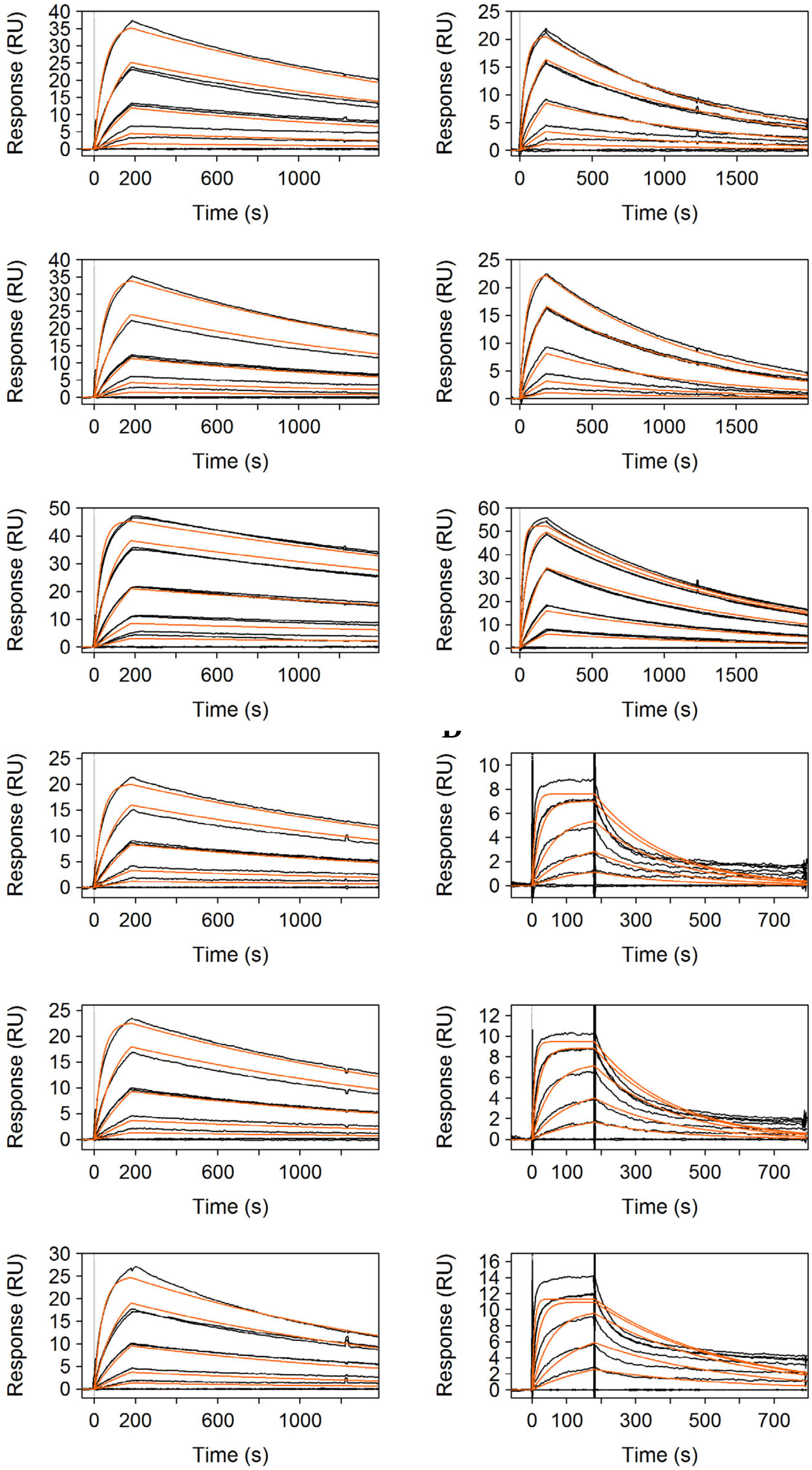

SPR-measurement: Kinetic profiles of receptors binding to captured Mabs/TsAbs;
A cMet, B HER3,C EGFR, D IGF1R.
